# Genetic Diversity and Relationship of Shanlan Upland Rice Were Revealed Based on 214 Upland Rice SSR Markers

**DOI:** 10.3390/plants12152876

**Published:** 2023-08-05

**Authors:** Rongju Li, Yinling Huang, Xinsen Yang, Meng Su, Huaiyang Xiong, Yang Dai, Wei Wu, Xinwu Pei, Qianhua Yuan

**Affiliations:** 1 College of Tropical Agriculture and Forestry, Hainan University, Haikou 570228, China; lirongju196982@163.com (R.L.); ylinghuang@126.com (Y.H.); xinsenyang@126.com (X.Y.); mengsu11@126.com (M.S.); weiwu@hainanu.edu.cn (W.W.); 2 Hainan Guangling High-Tech Industrial Co., Ltd., Lingshui 572400, China; xionghuaiyang@sina.com (H.X.); glnygs@163.com (Y.D.); 3 Biotechnology Research Institute, Chinese Academy of Agricultural Sciences, Beijing 100081, China

**Keywords:** Shanlan upland rice, upland rice, SSR, genetic diversity, genetic relationship

## Abstract

Shanlan upland rice (*Oryza sativa* L.) is a unique upland rice variety cultivated by the Li nationality for a long time, which has good drought resistance and high utilization value in drought resistance breeding. To explore the origin of Shanlan upland rice and its genetic relationship with upland rice from other geographical sources, 214 upland rice cultivars from Southeast Asia and five provinces (regions) in southern China were used to study genetic diversity by using SSR markers. Twelve SSR primers were screened and 164 alleles (Na) were detected, with the minimum number of alleles being 8 and the maximum number of alleles being 23, with an average of 13.667. The analysis of genetic diversity and analysis of molecular variance (AMOVA) showed that the differences among the materials mainly came from the individuals of upland rice. The results of gene flow and genetic differentiation revealed the relationship between the upland rice populations, and Hainan Shanlan upland rice presumably originated from upland rice in Guangdong province, and some of them were genetically differentiated from Hunan upland rice. It can be indirectly proved that the Li nationality in Hainan is a descendant of the ancient Baiyue ethnic group, which provides circumstantial evidence for the migration history of the Li nationality in Hainan, and also provides basic data for the advanced protection of Shanlan upland rice, and the innovative utilization of germplasm resources.

## 1. Introduction

Upland rice, also known as aerobic rice, is an ecological type of rice [1]. In areas where water layers are difficult to preserve or rice fields are prone to drought, long-term natural selection has led to the gradual evolution of rice varieties adapted to dryland cultivation [2]. Upland rice generally has a well-developed root system and other unique drought-resistant mechanisms [3] that can be cultivated in completely upland conditions, so it is more drought-tolerant than irrigated rice [4,5]. Shanlan upland rice (*Oryza sativa* L.) is an ancient local upland rice variety in Hainan province (China). It has the advantages of a short growth period, less water requirement, drought tolerance, and barren tolerance [6,7]. Most of the rice produced by Shanlan upland rice is japonica-glutinous rice with good quality and great taste [8]. More than 85% of the subspecies of Shanlan upland rice was of japonica type, which was closely related to the common wild rice in Guangdong (China) and Hunan (China). However, it is less closely related to the common wild rice in Hainan (China) and therefore has been speculated that it may originate from common wild rice in Guangdong (China) and Hunan (China) [9]. As the original parent material of rice breeding for drought resistance, Shanlan upland rice has highly elevated utilization value. In rice cross breeding, the development and utilization of favorable gene sources of Shanlan upland rice are worthy of full attention [10]. Upland rice has different adaptations and mechanisms in drought resistance compared to rice [11]. Therefore, hybrids may show greater drought resistance and adaptability. This makes the hybridization of rice and upland rice an essential genetic improvement mode [12,13] that can provide more options and support for agricultural production in arid areas. Therefore, the studying the genetic diversity and the genetic relationship of Shanlan upland rice can be used to search for better drought-resistant lines for hybrid rice breeding.

Simple sequence repeat (SSR) is a sequence of 1~6 nucleotides. Because of its high speed and specificity, it is often used as a rapid diagnostic tool [14]. Hinge et al. use SSR molecular markers as specific tags for banana species identification, which provides insights for further verification of the consistency of plant populations [15]. In recent years, SSR molecular markers have been widely used in animal and plant population analysis and plant genetic breeding [16,17,18]. Based on SSR molecular marker technique, Jasim et al. investigated the genetic diversity of aromatic rice germplasm. The results showed that 89% of the total variation of the germplasm came from within the population, while 11% of the variation came from the population [19]. Hassan et al. used 37 SSR markers to analyze the genetic diversity of 62 rice accessions in Kurdistan regions, indicating that 72% of the differences occurred between indica and japonica populations [20]. Liu et al. analyzed the genetic diversity of 1481 individuals using SSR markers and SNP haplotypes [21]. In addition, SSR also plays an essential role in the study of the genetic diversity of cowpea [22], barley [23], beans [24,25], maize [26], and other crops. Therefore, it is feasible to screen specific SSR primers for genetic diversity analysis of Shanlan upland rice to explore its geographical origin.

Li nationality is now recognized as the first ethnic group to move to Hainan (China). Scholars believe that the Li nationality ethnic group in Hainan originated in the northern mainland or Southeast Asia, but the most influential, and most scholars agree, is that the Li nationality came from the ancient Baiyue ethnic group. The primitive agriculture of Hainan Island began about 10,000 years ago in the Neolithic age. Modern archaeology can prove that the earliest residents of Hainan Island, the people of Luobi Cave, lived in Sanya (Hainan, China), where common wild rice, the ancestor of cultivated rice was distributed, but archaeological excavations have not found any evidence of rice remains. At present, the researches have shown that the relationship between Hainan Shanlan upland rice and Hainan common wild rice is relatively distantly, and Shanlan upland rice is not originating from the common wild rice in Hainan (China), but should be introduced from outside Hainan Island. The emergence and expansion of rice farming in China originated from the continuous migration of ancient Baiyue ethnic groups and “The rice growing techniques, rice culture, and rice seeds spread together” [27]. So it is worth studying where Shanlan upland rice was introduced to Hainan Island.

Therefore, we selected the original upland rice resources in the surrounding provinces of Hainan and Southeast Asia, and studied the origin and differentiation of upland rice and Shanlan upland rice by analyzing the genetic diversity and genetic relationship between upland rice and Shanlan upland rice in different geographical regions, to determine the possible geographical source of Shanlan upland rice in Hainan, providing basic data for further protecting Shanlan upland rice as an essential upland rice resource, as well as the innovative utilization of germplasm resources, and better excavation and utilization of Shanlan upland rice resources.

## 2. Results

### 2.1. Development of Polymorphic SSR Primers

We screened 48 pairs of primers from the rice database by gel electrophoresis, and 12 SSR primers were obtained for genetic diversity analysis (Table 1). A total of 164 alleles (Na) were detected at 12 SSR loci, with Na distribution ranging from 8 (LRJ89) to 23 (LRJ62) at each locus with an average Na of 13.667. The number of effective alleles (Ne) varied from 2.194 (LRJ89) to 7.579 (LRJ51), with a mean of 4.270. The Shannon Index (I) ranged from 1.136 (LRJ89) to 2.309 (LRJ65), with a mean of 1.728. Observed heterozygosity (Ho) ranged from 0.061 (LRJ53) to 0.151 (LRJ51), with a mean of 0.094; expected heterozygosity (He) ranged from 0.544 (LRJ89) to 0.868 (LRJ51), with a mean of 0.733. The fixed index (F) ranged from 0.826 (LRJ51) to 0.908 (LRJ81) with a mean of 0.873. The polymorphic information content (PIC) ranged from 0.513 (LRJ89) to 0.854 (LRJ51) with a mean of 0.704. Within-sample inbreeding coefficients (Fis) ranged from 0.811 (LRJ51) to 0.915 (LRJ88), with a mean of 0.872; total inbreeding coefficients (Fit) ranged from 0.815 (LRJ51) to 0.915 (LRJ88), with a mean of 0.875; The genetic differentiation coefficient (Fst) ranged from 0.008 (LRJ73) to 0.074 (LRJ49), with a mean of 0.028; and gene flow (Nm) ranged from 3.145 (LRJ49) to 32.639 (LRJ73), with a mean of 13.796.

### 2.2. Gene Flow among Upland Rice Populations from Different Geographical Origin

We analyzed the genetic diversity of 14 varieties of different geographic origins (Table 2), and the results showed that all populations of different geographic origins had gene flow greater than 1, indicating that the level of gene exchange among populations was relatively high between different geographic populations, which might be caused by some artificial activities such as variety selection and introduction exchange among various groups. For example, the level of gene exchange between the Philippines and Malaysia is relatively high, indicating that inter-introduction between the two regions has led to gene exchange between the two different populations; the lowest levels of gene exchange between Guizhou (China) and Vietnam, which may be that geographic distance and less human activity between the two places, resulting in lower level of gene exchange between the two different populations. In the lower triangles of the table, the Fst of the germplasm resources in Vietnam and other areas were particularly elevated, ranging from 0.071 to 0.119, indicating extreme genetic differentiation between these populations. The Fst of Hainan (China), and Hunan (China) was the lowest among inland germplasm (0.038), followed by Guangdong (China), Guangxi (China) and Guangdong (China) with an Fst of 0.032; the Fst of germplasm resources in five provinces of southern China ranged from 0.032 to 0.064. The results of minimal Fst and minimal genetic distance were consistent.

### 2.3. Genetic Diversity of Upland Rice from Different Geographical Sources

We analyzed the genetic diversity of 214 materials from 14 different geographical sources (Table 3). The results showed that Laos had the most Na (7.917) and Vietnam had the least Na (2.333). The Philippines has a maximum Ne of 4.512 and Vietnam has a minimum Ne of 1.952. Indonesia has the highest NE (1.625), indicating higher biodiversity in this region. The Ho (0.012) and He (0.713) of Myanmar were the lowest, indicating that the genetic variation was relatively large. In Hainan (China), Na (6.583), Ne (3.008), and I (1.268), were the lowest among 14 regions, Ho and He were 0.076 and 0.630, respectively, which was the intermediate level overall. Fst is an index of population differentiation determined by population genetic structure [28], the range of values is 0~1. A maximum value of 1 that there is complete genetic differentiation among populations, and 0, while 0 indicates no differentiation between populations. All Fst values in the table are relatively high (>0.7), indicating a significant degree of genetic differentiation between populations.

### 2.4. Genetic Similarity Analysis of Upland Rice from Different Geographical Sources

**Figure 1:**
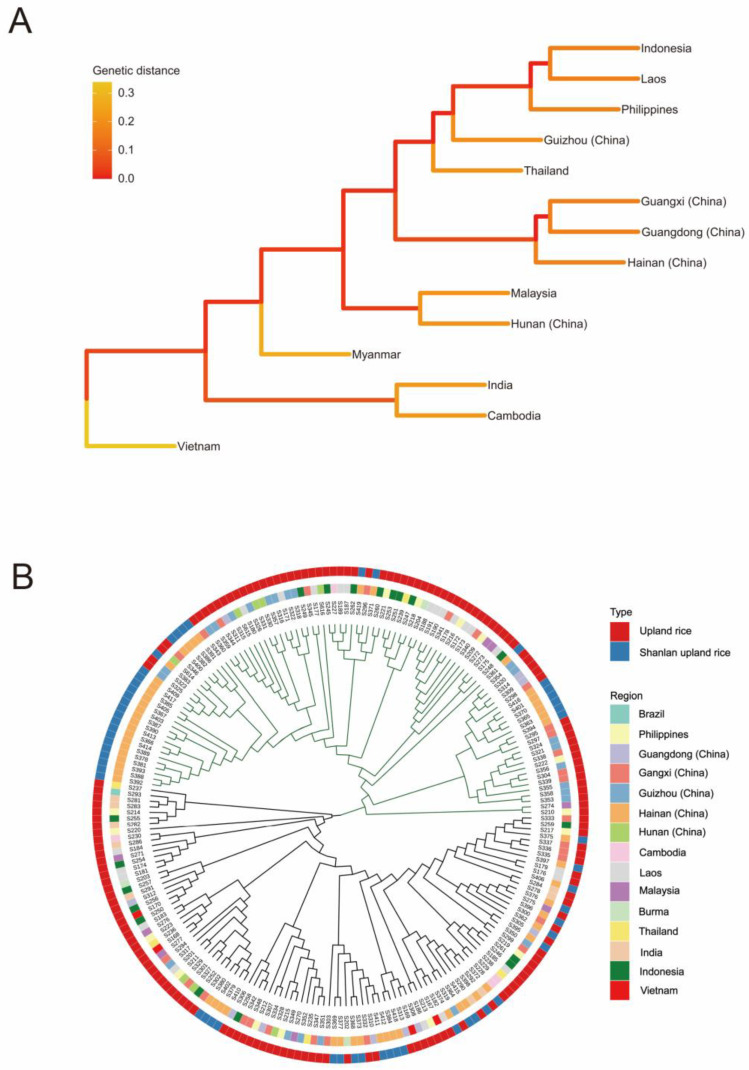
Genetic similarity analysis of upland rice from different geographical sources. (**A**) Genetic distance between populations in different regions. (**B**) UPGMA clustering results of 14 populations.

The Nei`s genetic distance between populations was calculated in POPGEN 1.3.2 [29]. The results (Figure 1A) showed that the genetic distance between populations ranged from 0.160 to 0.437, with an average genetic distance of 0.258, indicating that the genetic basis of germplasm resources was relatively abundant. Among the 14 populations, the genetic distance between Guizhou (China) and Vietnam was the largest (0.437), indicating that the two germplasm were distant from each other, while the genetic distance between the two populations was the smallest in Guangdong (China) and Guangxi (China) (0.160), indicating that the two germplasms were closely related. The genetic distance between Hainan (China) and Guangdong (China) was the closest at 0.165, followed by Guangxi (China) at 0.166 and Guizhou (China) at 0.177. Cluster analysis using Nei`s genetic distance-based unweighted group average method (UPGMA) (Figure 1B) showed that Guangxi (China) and Guangdong (China) were the most closely clustered of the 14 regional populations. The results indicated that Shanlan upland rice in Hainan (China) was closely related to upland rice in Guangdong (China) and Guangxi (China). The genetic distance analysis between Hainan (China) and Guangxi (China), Guangdong (China), Hunan (China), and Guizhou (China) showed that the genetic distance between Hainan (China) and Hunan (China) was 0.191, the genetic distances between Hunan (China) and Guangxi (China), Guangdong (China) and Guizhou (China) was between 0.2 and 0.3, indicating that the genetic relationship between Shanlan upland rice in Hainan (China) and upland rice in Hunan (China) was also closely related. The genetic distance of Hainan (China), Myanmar, and Vietnam was the most distant, with the genetic distance of 0.321 and 0.320, respectively.

### 2.5. Analysis of Molecular Variance

We used the AMOVA tool to explore genetic variation in 14 upland rice populations. The results showed that 4.34% of genetic variation existed between populations and about 95% of genetic variation existed in 214 upland rice germplasm. There was small genetic differentiation between populations (0.043, *p* < 0.001), which indicated that the gene mobility between populations was higher. The higher population Fis (0.871) indicated a lower genetic diversity within a population. In general, individual variation was the main source of total variation in upland rice samples (Table 4).

### 2.6. Cluster Analysis and Principal Coordinate Analysis of Upland Rice from Different Geographical Sources

**Figure 2:**
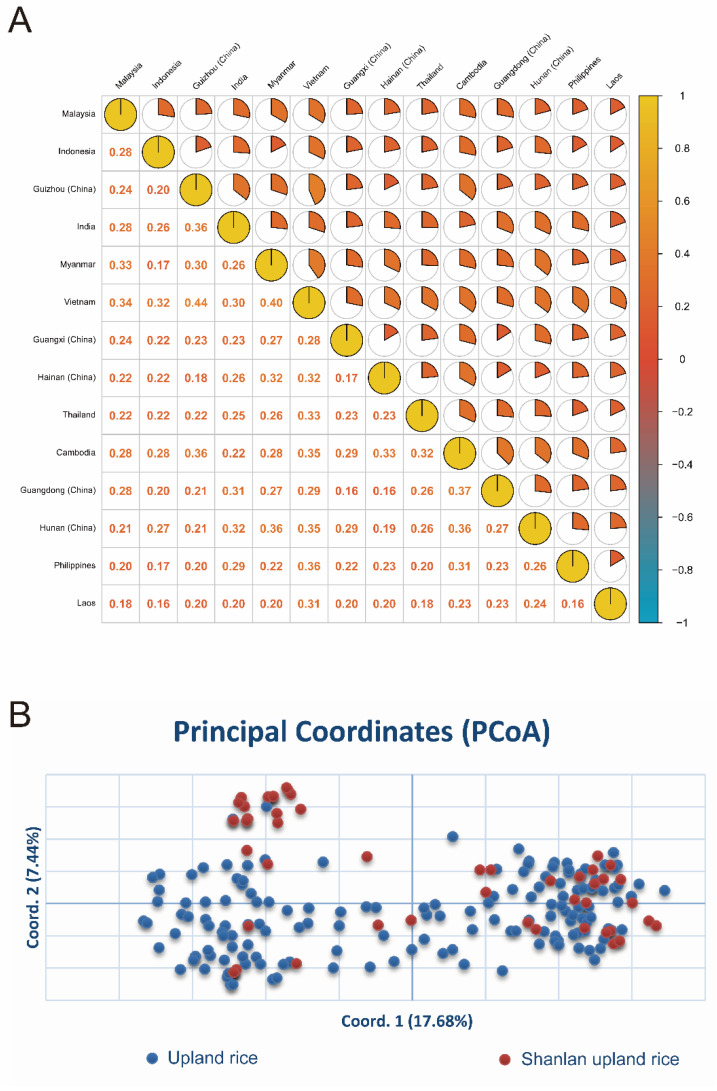
Cluster analysis and principal coordinate analysis of upland rice from different geographical sources. (**A**) Cluster Analysis of 214 Upland Rice Germplasm Resources. (**B**) Principal coordinates Analysis of 214 Rice Germplasm Resources.

To further analyze the genetic relationship among the populations, UPGMA clustering of the Nei’s genetic distance revealed that all populations were divided into two major branches (Figure 2A). The 214 materials were clustered into two major groups. The first group (green branches) included 30 Shanlan upland rice, of which 17 samples were clustered together separately, indicating that they were close or homologous. The genetic distances of 214 materials were analyzed by principal coordinate analysis (PCoA). The variation rates of Nei’s in horizontal and vertical coordinates were 17.68% and 7.44%, respectively. The two principal axes explained 25.12% of the total genetic variation. The materials were divided into two groups by PCoA (Figure 2B). The part of Shanlan upland rice in the first group was separated from upland rice, indicating that the genetic distance between Shanlan upland rice and upland rice was far, and the relationship between Shanlan upland rice was close or homologous. Another part of upland rice group includes Shanlan upland rice, indicating that the genetic distance between Shanlan upland rice and upland rice in this group is relatively close, which may be the result of inter-regional introduction. The UPGMA clustering results were consistent with the principal coordinate analysis, and all materials were classified into two groups.

## 3. Discussion

As a representative of the traditional farming culture of the ethnic minorities in Hainan, Shanlan upland Rice is an important genetic resource for food and agriculture. It is of great significance to study the origin and differentiation of Shanlan upland rice for species conservation and utilization. SSR is closely related to species evolution [30] and has become a powerful tool for studying species evolution and genetic variation [31]. In this study, we screened 12 SSR loci for genetic diversity analysis in 214 materials. The mean values of He and PIC of 12 loci were 4.297 and 0.703, respectively, and the polymorphism of these loci were higher than that research of Jasim et al. [19] in the study of genetic diversity of aromatic rice. Our mean values of Na (13.667) and I (1.728) were higher than those reported by Yang et al. [32] in Shanlan upland rice. PIC was able to measure the polymorphism of primers, and our PIC was higher than reported in other rice [33,34]. In general, our loci are highly polymorphic and can be used to study the genetic diversity of upland rice resources.

In the AMOVA-based analysis, we found that genetic diversity of 14 populations was mainly inter-individual. Environmental factors can influence the genetic variation of crops. Upland rice is a kind of cultivated rice that can adapt to drought stress and aerobic conditions. It is a crop which has evolved by long-term selection in dry land without water layer [7]. Crops evolved by long-term selection in drylands without aquifers. Environmental conditions affect plant growth and reproduction, affecting its genetic variation and adaptability [35,36]. The 214 materials were all upland rice, so the same environmental conditions, such as drought, may prompt them to evolve in the same direction, so there wasn’t much difference between the two groups. However, since rice is a self-pollinating crop, even the same variety under the same geographical conditions may produce different traits, and the differences in these traits may be gradually enlarged in long-term environmental effects or artificial selection, resulting in large differences between individuals. Therefore, in the process of rice genetic improvement, we should pay more attention to the excellent individuals in the population than to the selection of the population.

The study of genetic diversity can accurately reveal the evolutionary history of different species or the same population, allowing us to analyze and compare in greater depth the specific potential between different groups of different or the same species and the evolutionary direction of species or populations [37]. In our results, Hainan (China) and Hunan (China) had the largest Nm (6.343), followed by Guangdong (China) (5.985). Hainan (China) and Hunan (China) had the smallest coefficient of genetic differentiation (0.038), followed by Guangdong (China) (0.040). Genetic distance: Hainan (China) and Guangdong (China) were recently 0.165, followed by Guangxi (China) (0.166) and Guizhou (China) (0.177). The genetic distance between Hainan (China) and Hunan (China) was 0.191. In general, Shanlan upland rice seems to be more closely related to the resources in Guangdong (China) and Hunan (China). Guangdong (China) is the closest province to Hainan (China) in terms of geographical location. The clustering results of 214 materials were divided into two types: part of Shanlan upland rice was a single cluster and the remaining was a dispersed cluster. The analysis of genetic diversity showed that the genetic diversity of Hainan (China) Shanlan upland rice was at the middle level, this means that there are large genetic differences between individuals in a population. In addition, the analysis of molecular variance also showed that the 214-score material was mainly individual differences. These results indicated that different individuals in the Shanlan upland rice population were affected differently by external factors. It is mainly upland rice from Guangdong and Hunan (China).

Baiyue refers to the area where the ancient Yue people were distributed in the coastal area of southern China in ancient times. “The Li nationality in Hainan originated from the Baiyue people”, that is, Li nationality was split from one of the Baiyue’s ethnic groups, which is widely supported by Chinese classical philologists, archaeologists, geneticists, and anthropologists, and ethnologists [38]. However, there is no ancient written record of this claim, and there is also a lack of archaeological evidence. To this end, we selected the original upland rice resources from the surrounding provinces of Hainan (China) and Southeast Asia, and analyzed the genetic diversity and genetic relationship between upland rice and Shanlan upland rice, to determine the possible geographical origin of Shanlan upland rice in Hainan (China). Through the analysis of the genetic differentiation coefficient and genetic distance, we found that Shanlan upland rice from Hainan (China) is closely related to Guangdong (China) and Hunan (China) upland rice, however, the relationship between Hainan (China) and Hunan (China) is far from relationship with other countries in Southeast Asia, and the source of Hainan (China) is possibly from Guangdong (China) and Hunan (China). Guangdong is the province geographically closest to Hainan, and the southern Hunan upland rice growing area in southern China borders Guangdong and Guangxi, belonging to Baiyue (Nanyue). It can be indirectly proved that the Li nationality in Hainan (China) originated from a branch of Baiyue rather than from Southeast Asia, which provides circumstantial evidence for the migration history of the Li nationality people in Hainan (China).

## 4. Materials and Methods

### 4.1. Experimental Materials and DNA Extraction

A total of 214 materials were selected for the study, of which 55 were Shanlan upland rice of Hainan (China) and 159 from other geographical upland rice (Supplementary File S001). The seeds were sown in the experimental field of “Changshuiyang base”, Lingshui, Hainan (China), located at latitude 18°29′39.70″ N and longitude 110°02′30.95″ E, belonging to the tropical monsoon island-type climate. The fresh leaves of 3 randomly selected plants were stored in liquid nitrogen for quick freezing, DNA was extracted from leaves with a magnetic beads kit (NMG2611-96, Wuhan Nano Magnetic Biotechnology Co., Ltd., Wuhan, China) and stored at −20 °C. The concentration and purity of DNA were detected with NanoDROP 8000 photometer.

### 4.2. Primer Screening and Genetic Diversity Analysis

Forty-eight pairs of primers were selected from the rice database and 21 BP (GAAGGTGACCAAGTTCATGCT) linker sequence was added to the upstream sequence of the primers. To screen primers, 17 varieties from 15 regions were used for PCR amplification, and 12 polymorphic primers were selected for population typing of 214 rice (Supplementary File S002). Then we performed fluorescent PCR, diluted the product to 10–20 ng/uL, and configured the machine system (1 μL fluorescent PCR product; 0.5μL GeneScan™500 LIZ; 8.5 μL Hi-Di™ Formamide, run the SSR sample analysis assay on the ABI 3730xl.

The original genotype data derived from ABI 3730XL were then transferred into GenAlEx version 6.501 software [39] to calculate the various genetic diversity indicators of SSR loci and populations, the observed allele (Na), effective allele (Ne), Shannon index (I), polymorphism information index (PIC), observed heterozygosity (Ho), expected heterozygosity (He) and inbreeding coefficient (Fis) were included.

### 4.3. Molecular Variance Analysis (AMOVA) and Gene Flow Estimation

The original genotype data of Shanlan upland rice and upland rice were used to calculate the variation, differentiation, and significance test in GenAlEx version 6.501 software. Gene flow (*Nm*) was calculated based on the genetic differentiation coefficient (Fst) obtained from GenAlEx version 6.501 [40]:

Nm=0.25(1−Fst)∕Fst

### 4.4. Data Processing

Data were collated and analyzed using Excel 2019, and UPGMA clustering and mapping were performed by using R (version 4.0.0). The Nei’s genetic distances (Supplementary Files S003 and S004) were clustered in R using the “hclust”. Tree graphs are drawn using the R package “ggtree”.

## 5. Conclusions

In this study, SSR markers were used to analyze the genetic diversity of Shanlan upland rice. 12 pairs of polymorphic primers were screened from 214 materials from different regions. In our results, we found that the genetic differences of upland rice in different regions mainly existed between individuals, and the population of Shanlan upland rice from Hainan (China) also showed different genetic differences, and the genetic diversity analysis showed that it may came from rice in Guangdong (China) and Hunan (China). This study provides some theoretical support for further exploration of the Li nationality origin from the perspective of rice seed source.

## Figures and Tables

**Table 1:** 12 pairs of primers diversity analysis.

Locus	Na	Ne	I	Ho	He	F	PIC	Fis	Fit	Fst	Nm
LRJ49	12.000	3.947	1.652	0.084	0.747	0.887	0.712	0.869	0.879	0.074	3.145
LRJ50	10.000	4.162	1.624	0.093	0.760	0.877	0.721	0.885	0.892	0.058	4.039
LRJ51	16.000	7.579	2.241	0.151	0.868	0.826	0.854	0.811	0.815	0.025	9.834
LRJ53	20.000	2.499	1.578	0.061	0.600	0.898	0.585	0.897	0.898	0.012	21.093
LRJ54	16.000	3.814	1.719	0.094	0.738	0.873	0.704	0.869	0.873	0.031	7.748
LRJ62	23.000	5.830	2.194	0.099	0.828	0.881	0.809	0.874	0.877	0.030	8.002
LRJ65	17.000	6.989	2.309	0.113	0.857	0.868	0.845	0.877	0.881	0.031	7.938
LRJ71	11.000	4.396	1.790	0.123	0.772	0.841	0.747	0.838	0.840	0.009	26.146
LRJ73	11.000	3.353	1.552	0.098	0.702	0.860	0.663	0.860	0.861	0.008	32.639
LRJ81	9.000	3.451	1.499	0.065	0.710	0.908	0.666	0.904	0.907	0.030	7.971
LRJ88	11.000	3.030	1.447	0.066	0.670	0.902	0.625	0.915	0.915	0.009	26.846
LRJ89	8.000	2.194	1.136	0.079	0.544	0.854	0.513	0.867	0.870	0.024	10.149
Mean	13.667	4.270	1.728	0.094	0.733	0.873	0.704	0.872	0.876	0.028	13.796

Note: Na: Alleles. Ne: Effective alleles. I: Shannon Index. Ho: Observed heterozygosity. He: Expected heterozygosity. F: Fixed index. PIC: Polymorphic information content. Fis: Inbreeding coefficients. Fit: Total inbreeding coefficients. Fst: Genetic differentiation coefficient. Nm: Gene flow.

**Table 2:** Gene flow (upper triangle) and genetic differentiation coefficient (lower triangle) between populations.

	Philippines	Guangdong (China)	Guangxi (China)	Guizhou (China)	Hainan (China)	Hunan (China)	Cambodia	Laos	Myanmar	Malaysia	Thailand	India	Indonesia	Vietnam
Philippines	-	6.302	6.932	5.698	4.766	4.518	3.394	12.174	6.668	7.35	6.356	3.678	10.337	2.103
Guangdong (China)	0.038	-	7.58	5.064	5.958	4.349	2.656	5.489	5.201	3.887	4.883	3.006	7.396	2.327
Guangxi (China)	0.035	0.032	-	3.477	5.353	3.66	3.881	6.969	5.2	4.664	6.306	4.402	7.241	3.278
Guizhou (China)	0.042	0.047	0.067	-	5.086	5.004	1.952	5.327	3.784	3.497	3.973	2.153	5.919	1.298
Hainan (China)	0.05	0.04	0.045	0.047	-	6.343	2.412	4.657	3.375	4.059	4.465	3.586	5.133	1.79
Hunan (China)	0.052	0.054	0.064	0.048	0.038	-	2.177	4.167	3.191	3.889	4.225	2.873	4.122	1.64
Cambodia	0.069	0.086	0.061	0.114	0.094	0.103	-	4.905	4.312	2.924	4.008	5.595	4.069	2.131
Laos	0.02	0.044	0.035	0.045	0.051	0.057	0.048	-	7.699	6.81	9.764	4.796	12.376	2.368
Myanmar	0.036	0.046	0.046	0.062	0.069	0.073	0.055	0.031	-	3.555	5.75	3.904	10.075	1.954
Malaysia	0.033	0.06	0.051	0.067	0.058	0.06	0.079	0.035	0.066	-	5.14	3.169	4.499	1.843
Thailand	0.038	0.049	0.038	0.059	0.053	0.056	0.059	0.025	0.042	0.046	-	4.643	7.31	2.538
India	0.064	0.077	0.054	0.104	0.065	0.08	0.043	0.05	0.06	0.073	0.051	-	3.894	2.183
Indonesia	0.024	0.033	0.033	0.041	0.046	0.057	0.058	0.02	0.024	0.053	0.033	0.06	-	2.577
Vietnam	0.106	0.097	0.071	0.161	0.123	0.132	0.105	0.096	0.113	0.119	0.09	0.103	0.088	-

**Table 3:** Genetic diversity among populations.

Sample Plot		Na	Ne	I	Ho	He	F
Philippines	Mean	6.583	4.512	1.621	0.032	0.754	0.957
	SE	0.543	0.449	0.092	0.011	0.024	0.014
Guangdong	Mean	4.500	3.248	1.276	0.039	0.669	0.942
(China)	SE	0.337	0.265	0.080	0.012	0.027	0.018
Guangxi	Mean	5.833	3.501	1.400	0.089	0.674	0.876
(China)	SE	0.575	0.359	0.107	0.015	0.038	0.019
Guizhou	Mean	7.333	3.729	1.482	0.162	0.684	0.768
(China)	SE	0.829	0.492	0.125	0.021	0.034	0.025
Hainan	Mean	6.583	3.008	1.268	0.076	0.630	0.882
(China)	SE	0.657	0.296	0.095	0.011	0.036	0.012
Hunan	Mean	4.167	3.055	1.145	0.131	0.613	0.789
(China)	SE	0.588	0.420	0.122	0.027	0.046	0.041
Cambodia	Mean	4.167	3.092	1.158	0.031	0.592	0.945
	SE	0.423	0.374	0.148	0.016	0.071	0.028
Laos	Mean	7.917	4.223	1.556	0.081	0.704	0.887
	SE	0.933	0.688	0.137	0.014	0.041	0.016
Myanmar	Mean	4.833	3.978	1.427	0.012	0.713	0.983
	SE	0.322	0.424	0.091	0.012	0.033	0.017
Malaysia	Mean	4.417	3.398	1.282	0.205	0.668	0.702
	SE	0.379	0.344	0.097	0.032	0.036	0.045
Thailand	Mean	4.750	3.546	1.307	0.075	0.656	0.878
	SE	0.494	0.399	0.135	0.028	0.055	0.049
India	Mean	4.917	3.076	1.193	0.072	0.588	0.876
	SE	0.596	0.372	0.156	0.015	0.069	0.031
Indonesia	Mean	7.250	4.466	1.625	0.067	0.740	0.907
	SE	0.592	0.537	0.103	0.014	0.029	0.022
Vietnam	Mean	2.333	1.952	0.714	0.042	0.453	0.860
	SE	0.142	0.157	0.072	0.028	0.040	0.101
Total	Mean	5.399	3.485	1.318	0.080	0.653	0.875
	SE	0.186	0.118	0.034	0.006	0.013	0.011

Note: Na: Alleles. Ne: Effective alleles. I: Shannon Index. Ho: Observed heterozygosity. He: Expected heterozygosity. Fst: Genetic differentiation coefficient.

**Table 4:** Analysis of Molecular Variance of 214 Upland Rice populations.

Source	df	SS	MS	Est. Var.	PV%	Fst	Fis	Fit
Among Pops	1	40.418	40.418	0.197	4.340%			
Among Indiv	212	1726.285	8.143	3.791	83.333%			
Within Indiv	214	120.000	0.561	0.561	12.326%			
Total	427	1886.703		4.549	100%	0.043 ***	0.871 ***	0.877 ***

Note: Source: variation. df: Degree of freedom. SS: Total variance. MS: Mean square variance; EST. Var.: Estimated variance. PV%: Percent variation. Fst: Genetic differentiation coefficient. Fis: Inbreeding coefficient. Fit: total inbreeding coefficients. *** indicates significant difference *p* < 0.001.

## Data Availability

Data is contained within the article or Supplementary Materials.

## Supplementary Materials

The following supporting information can be downloaded at: https://www.mdpi.com/article/10.3390/plants12152876/s1, Supplementary File S001: A brief description of 214 materials; Supplementary File S002: Primers for genetic diversity analysis; Supplementary File S003: Genetic distance of 14 regions; Supplementary File S004: Genetic distance of 214 materials.
